# Interspecies transmission of porcine-originated G4P[6] rotavirus A between pigs and humans: a synchronized spatiotemporal approach

**DOI:** 10.3389/fmicb.2023.1194764

**Published:** 2023-05-22

**Authors:** Valentina Kunić, Tina Mikuletič, Rok Kogoj, Tom Koritnik, Andrej Steyer, Silvija Šoprek, Goran Tešović, Vlatka Konjik, Ivana Roksandić Križan, Marina Prišlin, Lorena Jemeršić, Dragan Brnić

**Affiliations:** ^1^Virology Department, Croatian Veterinary Institute, Zagreb, Croatia; ^2^School of Medicine, Institute for Microbiology and Immunology, University of Ljubljana, Ljubljana, Slovenia; ^3^Public Health Microbiology Department, National Laboratory of Health, Environment, and Food, Ljubljana, Slovenia; ^4^Department for Pediatric Infectious Diseases, University Hospital for Infectious Diseases “Dr. Fran Mihaljević”, Zagreb, Croatia; ^5^School of Medicine, University of Zagreb, Zagreb, Croatia; ^6^Clinical Hospital Center Osijek, Osijek, Croatia

**Keywords:** rotavirus A, human, zoonosis, domestic pig, G4P[6], reassortment, recombination, Croatia

## Abstract

As a leading viral cause of acute gastroenteritis in both humans and pigs, rotavirus A (RVA) poses a potential public health concern. Although zoonotic spillover of porcine RVA strains to humans is sporadic, it has been detected worldwide. The origin of chimeric human–animal strains of RVA is closely linked to the crucial role of mixed genotypes in driving reassortment and homologous recombination, which play a major role in shaping the genetic diversity of RVA. To better understand how genetically intertwined porcine and zoonotic human-derived G4P[6] RVA strains are, the present study employed a spatiotemporal approach to whole-genome characterization of RVA strains collected during three consecutive RVA seasons in Croatia (2018–2021). Notably, sampled children under 2 years of age and weanling piglets with diarrhea were included in the study. In addition to samples tested by real-time RT-PCR, genotyping of VP7 and VP4 gene segments was conducted. The unusual genotype combinations detected in the initial screening, including three human and three porcine G4P[6] strains, were subjected to next-generation sequencing, followed by phylogenetic analysis of all gene segments, and intragenic recombination analysis. Results showed a porcine or porcine-like origin for each of the eleven gene segments in all six RVA strains. The G4P[6] RVA strains detected in children most likely resulted from porcine-to-human interspecies transmission. Furthermore, the genetic diversity of Croatian porcine and porcine-like human G4P[6] strains was propelled by reassortment events between porcine and porcine-like human G4P[6] RVA strains, along with homologous intragenotype and intergenotype recombinations in VP4, NSP1, and NSP3 segments. Described concurrent spatiotemporal approach in investigating autochthonous human and animal RVA strains is essential in drawing relevant conclusions about their phylogeographical relationship. Therefore, continuous surveillance of RVA, following the One Health principles, may provide relevant data for assessing the impact on the protectiveness of currently available vaccines.

## 1. Introduction

Rotavirus A (RVA) group is continuously reported as a leading cause of non-bacterial gastroenteritis in mammal and avian species, especially offspring. In humans, it can infect neonates, older children, and sometimes adults, with children younger than 5 years being the most affected (Trojnar et al., 2009; Estes and Greenberg, 2013; Desselberger, 2014). Global RVA mortality burden started decreasing after the early 2000s, counting more than 250,000 deaths, to estimated 128,500 deaths in 2016 as more countries introduced vaccines into their National Immunization Programs (NIP) (Tate et al., 2016; Troeger et al., 2018). The most common symptoms associated with RVA-induced acute gastroenteritis (AGE) typically include profuse diarrhea, vomiting, and fever. The need for hospital care often stems from dehydration and reduced ability for oral intake (Dennehy, 2008; Crawford et al., 2017). RVAs are also a major causative agent of viral AGE in pigs, mainly in suckling and weaned pig age groups, causing substantial financial costs to the pork industry (Chang et al., 2012). *Rotavirus A* species belongs to the *Rotavirus* genus within the *Reoviridae* family, whose genome consists of double-stranded RNA arranged in 11 genome segments. The VP7 and VP4 segments are the basis for the binomial nomenclature of rotaviruses, providing the G and P genotypes, respectively (Estes and Greenberg, 2013). However, whole-genome-based classification has been developed and increasingly used in recent years (Maunula and von Bonsdorff, 2002). The respective genotypes are assigned to each genomic segment based on the percentage identity cutoff values for nucleotide (nt) coding sequences of each viral (VP) and non-structural protein (NSP) (Matthijnssens et al., 2008a). This whole-genome classification aims to detect the genetic relationships between RVAs derived from different host species, reassortment events, and previously undetected genotypes (Matthijnssens et al., 2008b). Reassortment and recombination events are driving rotavirus diversification, which sometimes results in the emergence of chimeric human-animal strains. It is well-known that some RVA genotypes are more common in certain species, and many of them are shared between different species (Martella et al., 2010; McDonald et al., 2016). The human Wa-like and porcine RVAs are considered to have a common origin source since genogroup 1 genes found in the human RVA strains with the Wa-like constellation (i.e., I1-R1-C1-M1-A1-N1-T1-E1-H1) are also frequently found in porcine RVA strains (Matthijnssens et al., 2008b; Papp et al., 2013a; Theuns et al., 2015; Silva et al., 2016). Moreover, certain G/P genotype combinations can be considered usual or unusual for the given species. Therefore, the G4P[6] genotype combination is considered an unusual combination in humans, but it is quite common in pigs (Doro et al., 2015). Detection of a rare genotype combination like this one in a secondary host species may indicate a recent interspecies transmission event. In such cases, whole-genome sequencing can be used as a method of choice for strain investigation (Doro et al., 2015). Even though it is considered unusual in the human population, a G4P[6] genotype was discovered to reappear globally (Tacharoenmuang et al., 2021). A previous epidemiological study about the occurrence of RVA genotypes in children in Croatia reports a single case of the G4P[6] genotype (Vrdoljak et al., 2019). In our recent study on RVAs circulating in domestic pigs and wild boars, G4P[6] combination showed its modest appearance in domestic pigs, with the overall prevalence of G4 and P[6] strains among genotyped samples of only 9.8 and 4.3%, respectively (Brnić et al., 2022).

The present study aimed to comparatively analyze whole genomes of G4P[6] RVA strains detected in symptomatic children and pigs in Croatia with the synchronized spatiotemporal approach. It offers an insight into G4P[6] RVAs circulating in both populations during the same timeframe and relatively small geographical region, giving an opportunity for drawing adequate conclusions on the possible interspecies transmission, reassortment, and intragenic recombination events, which individually and collectively boost RVA genetic diversity in Croatian ecosystem.

## 2. Material and methods

### 2.1. Sampling

Stool samples and rectal swabs were collected from RVA-infected children and domestic pigs, respectively, which were sampled from 2018 to 2021, accounting for RVA seasons 2018/2019, 2019/2020, and 2020/2021. Sampling was conducted continuously, comprising rotavirus in-season and out-of-season samples. Mostly, children under 5 years of age with present clinical signs of acute gastroenteritis, consequently admitted to the University Hospital for Infectious Diseases “Dr. Fran Mihaljević” Zagreb and Clinical Hospital Center Osijek, were included in this study. The collected stool samples were initially tested for the presence of rotaviral and adenoviral antigens using a single commercial immunochromatographic assay, the Rota-AdenoGnost (BioGnost, Zagreb, Croatia). During the same timeframe, the piglets with or without diarrhea were sampled, each by individual rectal swabbing, at large industrial and small backyard holdings in multiple counties as described in our recent work (Brnić et al., 2022). The piglets whose samples are reported in this research showed clinical signs of acute gastroenteritis at the time of sampling. Collected stool and swab samples were transferred to the Croatian Veterinary Institute for subsequent laboratory testing, maintaining a cold chain while in transportation. The samples were further processed immediately after reception or stored at −80°C. Detailed information about the sampled individuals is shown in Table 1.

**Table 1:** Data about human and domestic pig samples included in the present study.

**RVA strain ID**	**Gender**	**Age**	**Sampling time**	**Diarrhea**	**Vesikari score**	**RVA Vaccine**	**County**	**Country**
RVA/Human-wt/HRV/D230-ZG/2019/G4P[6]	f	1 y and 10 m	August/2019	yes	11	No	City of Zagreb	Croatia
RVA/Human-wt/HRV/D329-OB/2019/G4P[6]	f	1 y and 9 m	July/2019	yes	ND^*^	No	Osijek-Baranja	Croatia
RVA/Human-wt/HRV/D572-ZG/2021/G4P[6]	f	1 y and 4 m	July/2021	yes	9	No	City of Zagreb	Croatia
RVA/Pig-wt/HRV/S243-VS/2019/G4P[6]	m	30–40 days	November/ 2019	yes	N/A^*^	No	Vukovar-Srijem	Croatia
RVA/Pig-wt/HRV/S338-Z/2020/G4P[6]	f	37 days	March/2020	yes	N/A^*^	No	Zagreb	Croatia
RVA/Pig-wt/HRV/S344-Z/2020/G4P[6]	f	37 days	March/2020	yes	N/A^*^	No	Zagreb	Croatia

^*^ND–not determined since a child was not hospitalized. She was admitted to the pediatric ambulatory care unit, with symptoms resolved after 2 days. ^*^N/A–not applicable.

### 2.2. Molecular diagnostics

Molecular diagnostics including RNA extraction, VP2 real-time RT-PCR, VP4, and VP7 genotyping were conducted within the scope of the initial screening of samples and are described in our study on RVAs in domestic pigs and wild boars (Brnić et al., 2022). For the human samples, the exception was the VP4/VP7 genotyping which was performed with the application of a multiplex VP7 and VP4 RT-PCR (EuroRotaNet, 2009,^1^; Fujii et al., 2019) complemented with the Sanger sequencing of untypable strains. Based on genotyping results, six G4P[6] strains, three of human and three of porcine origin, were selected for next-generation sequencing (NGS).

### 2.3. Library preparation and NGS

Three individual sequencing runs were performed chronologically as samples were collected. Firstly, 20% *w/v* fecal and swab suspensions prepared with Medium 199 (Sigma-Aldrich, St. Louis, USA) were used as a starting material. Suspensions were sent to the Institute of Microbiology and Immunology, Slovenia, where sample preparation and NGS were conducted. Nucleic acid extraction from the supernatant of 20% *w/v* fecal and swab suspension was performed on a Maelstrom 9600 device (TANBead Inc., Taoyuan City, Taiwan) using an OptiPure Viral Auto Plate (TANBead Inc., Taoyuan City, Taiwan) extraction kit, followed by the real-time RT-PCR detection of RVA by LightMix Modular assay (TIB Molbiol, Berlin, Germany) on a LightCycler 480 instrument (Roche, Basel, Switzerland). Since viral genome loads in metagenomic samples tend to be exceptionally low in concentration, DNA depletion was performed using TURBO DNA-free™ Kit (Thermo Fisher Scientific, Waltham, USA). After DNA removal, the Maxima H Minus Double-Stranded cDNA Synthesis Kit (Thermo Scientific™, Waltham, USA) was used for the first- and second-strand cDNA synthesis. Prepared dsDNA was then purified utilizing GeneJET PCR Purification Kit (Thermo Fisher Scientific, Waltham, USA) to remove excess dNTPs and other reagents such as competing enzymes or buffer components. All procedures referenced above were performed following the respective manufacturer's instructions. Complementary DNA (cDNA) was finally quantified before proceeding with library preparation, using a Qubit™ 4 Fluorometer with a Qubit dsDNA HS Assay Kit (Thermo Fisher Scientific, Waltham, USA).

NGS libraries were constructed using a Nextera XT DNA Library Preparation Kit (Illumina Inc., San Diego, USA) with barcoding respective samples with the IDT^®^ for Illumina^®^ Nextera DNA/RNA Unique Dual Indexes Set B and C (Illumina Inc., San Diego, USA) according to the manufacturer's instructions.

After tagmentation and amplification, NGS libraries were purified using Agencourt AMPure XP magnetic beads (Beckman Coulter, Brea, USA). The quality and quantity of the purified libraries were assessed with a 2100 Bioanalyzer instrument (Agilent, Santa Clara, USA) using a High Sensitivity DNA Kit (Agilent, Santa Clara, USA), and a Qubit™ 4 Fluorometer using Qubit dsDNA HS Assay (Thermo Fisher Scientific, Waltham, USA), respectively. NGS was performed on lllumina^®^ NextSeq 500 sequencer (Illumina Inc., San Diego, USA) utilizing the NextSeq 500/550 High Output Kit v 2.5 on 300 cycles (Illumina Inc., San Diego, USA) to produce 150 paired-end reads. The herein-described procedure was applied for all three individual sequencing runs.

### 2.4. NGS data analysis

Data analysis for the NGS was performed using a CLC Genomics Workbench 22.0.2 (Qiagen, Hilden, Germany). For each of the 11 RVAs genomic segments, representative sequences of various genotypes were selected using NCBI's Virus Variation Rotavirus Database^2^ (Hatcher et al., 2017). Those were used for building reference lists for each gene segment, regardless of the genotype. Genomes were assembled utilizing the reference-based mapping process for each gene segment individually due to the segmented nature of the rotavirus genome. The workflow consisted of trimming raw reads of Illumina adapters, mapping trimmed reads to all the reference lists, and finally extracting consensus sequences and mapping reports. Consensus sequences were not considered for further investigation if they did not meet the previously defined minimum sequence length and identity criteria (Matthijnssens et al., 2008a) or distribution coverage of 90% and coverage depth of 10 × . Any occurring sequence gaps were managed by performing a *de novo* assembly on the same samples and correlating relevant contigs with the relevant reference-based consensus assemblies. Additional mapping data containing accession numbers of each genotype reference sequence used for the mapping process can be found in Supplementary Table 1 for each RVA strain characterized in the present study. Final consensus sequences for every gene segment prior to the genotyping process were selected based on the mapping quality and the consequent full-length consensus sequence completeness. Genotypes were confirmed using final consensus sequences as queries, in the BLASTn search tool^3^ in addition to the ViPR tool version 3.28.22^4^ (Pickett et al., 2011), and characterized following previously described guidelines defining genotype cutoff values (Matthijnssens et al., 2008a). During these searches, any consensus sequence that did not hold up to the respective genotype it was initially mapped to was herein discarded as a result of the mapping error. Strain names were assigned according to the RVA nomenclature uniformity guidelines administered by the Rotavirus Classification Working Group^5^ (RCWG). The CDSs that shared the highest percentage identity with each query or representatives of a certain group of sequences were used to assemble multiple sequence alignments and conduct evolutionary analyses in MEGA 11 software (Tamura et al., 2021).

### 2.5. Phylogenetic analysis and pairwise comparison

To investigate the evolutionary relationship between human and porcine RVA G4P[6] genotype strains, we constructed individual phylogenetic trees for each of the 11 RVA genomic segments, alongside the calculation of pairwise identity matrices. Therefore, we chose the representative strains from GenBank based on their high percentage identity with our query sequences and comparability based on geolocation, origin, host, or lineage for comparison purposes. The evolutionary history was inferred using the maximum-likelihood (ML) method for each multiple sequence alignment obtained by the MUSCLE algorithm, both acquired utilizing MEGA 11 software (Tamura et al., 2021). Substitution models that demonstrated the lowest BIC score values were as follows: T92+G (VP6, NSP2, NSP4, NSP5), T92+G+I (VP7, NSP1, NSP3), TN93+G+I (VP2), GTR+G+I (VP1, VP3), and HYK+G+I (VP4). The bootstrap analysis with 1,000 replicates was used to assess the branching support for each ML tree. Different G4 lineages were determined based on lineage attribution from Wandera et al. (2021). Different P[6] genotype lineages were determined based on the lineage attribution from Maringa et al. (2020) and Wandera et al. (2021). For graphical editing and annotation of phylogenetic trees, we used iTOL version 6^6^ (Letunic and Bork, 2021). Furthermore, CLC Genomics Workbench 22.0.2 (Qiagen, Hilden, Germany) was used for calculating pairwise identity matrices among the previously aligned RVA sequences from the GenBank and the strains from the present study (Supplementary Tables 2, 3). Obtained nt and amino acid (aa) sequences of complete CDS for each RVA gene segment, including additional genotypes in mixed infections, were submitted to the GenBank with adjacent accession numbers: D230: OQ440159-OQ440170; D329: OQ440171-OQ440184; D572: OQ440185-OQ440195; S243: OQ440196-OQ440210; S338: OQ440211-OQ440223; and S344: OQ440224-OQ440236 (Supplementary Table 4).

### 2.6. Intragenic recombination analysis

Utilizing the BLASTn tool, we identified and downloaded complete BLAST search results for each of the 11 genome segments of six G4P[6] Croatian strains, including their respective mixed genotypes where applicable. Multiple sequence alignment sets were constructed as described earlier, with the number of sequences analyzed per gene alignment ranging from 110 to 383. The RDP4 v.4.101 software was used to perform intragenotype (for each gene) and intergenotype (for genes with apparent mixed genotypes) recombination analysis by applying seven integrated recombination detection methods: RDP, GENECONV, MaxChi, Bootscan, Chimera, SiScan, and 3Seq (Martin et al., 2015). For every detected recombination event, the UPGMA method constructed the breakpoint-defined major and minor parent phylogenetic trees (data not shown). The term parent in this context does not point out the exact evolutionary progenitors of the recombinant strains, it rather signifies a group of RVA strains from which the actual progenitors might have originated. Only putative homologous recombination predicted by at least six program methods was considered positive recombination signals (Hoxie and Dennehy, 2020).

## 3. Results

### 3.1. NGS results and the whole-genome constellation of RVA strains

Illumina NextSeq 500 platform yielded 23.5 × 10^6^ reads (~122 bp average length), 18.1 × 10^6^ reads (~110 bp av. Length), 28.5 × 10^6^ reads (~110 bp av. length), 29.6 × 10^6^ reads (~115 bp av. length), 24.7 × 10^6^ reads (~147 bp av. length), and 16.8 × 10^6^ reads (~111 bp av. length) for strains D230, D329, S243, S338, S344, and D572, respectively. Complete coding sequences were successfully determined for all 11 gene segments of all sequenced strains, and their respective mixed genotypes, except for the P[13] genotype of the S243 strain, which was a partial CDS (97%). A number of mapped reads, average coverage, and other reference mapping-related data are summarized in Supplementary Table 1 for each reported RVA strain. Concatenated 11-gene segmented genome constellations are presented in Table 2. Croatian G4P[6] porcine-like human RVA strains displayed a Wa-like genogroup constellation, and porcine G4P[6] strains displayed an RVA genogroup 1 constellation. Each gene segment of both human and domestic pig RVA strains uncovered the evolutionary connection with porcine and porcine-like human strains from neighboring countries but also with some distant global RVA strains. Pairwise identity matrices for each gene segment can be found in Supplementary Table 2.

**Table 2:** Whole-genome constellations of six Croatian RVA G4P[6] strains.

**RVA strain ID**	**VP7**	**VP4**	**VP6**	**VP1**	**VP2**	**VP3**	**NSP1**	**NSP2**	**NSP3**	**NSP4**	**NSP5**
RVA/Human-wt/HRV/D230-ZG/2019/G4P[6]	G4	P[6]	I1	R1	C1	M1	A1	N1	T1/T7	E1	H1
RVA/Human-wt/HRV/D329-OB/2019/G4P[6]	G4/G1	P[6]/P[8]	I1	R1	C1	M1	A1	N1	T1/T7	E1	H1
RVA/Human-wt/HRV/D572-ZG/2021/G4P[6]	G4	P[6]	I1	R1	C1	M1	A8	N1	T7	E1	H1
RVA/Pig-wt/HRV/S243-VS/2019/G4P[6]	G4/G5/G11	P[6]/P[13]	I5	R1	C1	M1	A8	N1	T1/T7	E9	H1
RVA/Pig-wt/HRV/S338-Z/2020/G4P[6]	G4/G5	P[6]/P[13]	I5	R1	C1	M1	A1	N1	T7	E1	H1
RVA/Pig-wt/HRV/S344-Z/2020/G4P[6]	G4/G5	P[6]/P[13]	I5	R1	C1	M1	A1	N1	T7	E1	H1

Mixed genotypes are designated for the respective genomic segment.

### 3.2. Phylogenetic and recombination analysis

#### 3.2.1. VP7

**Figure 1:**
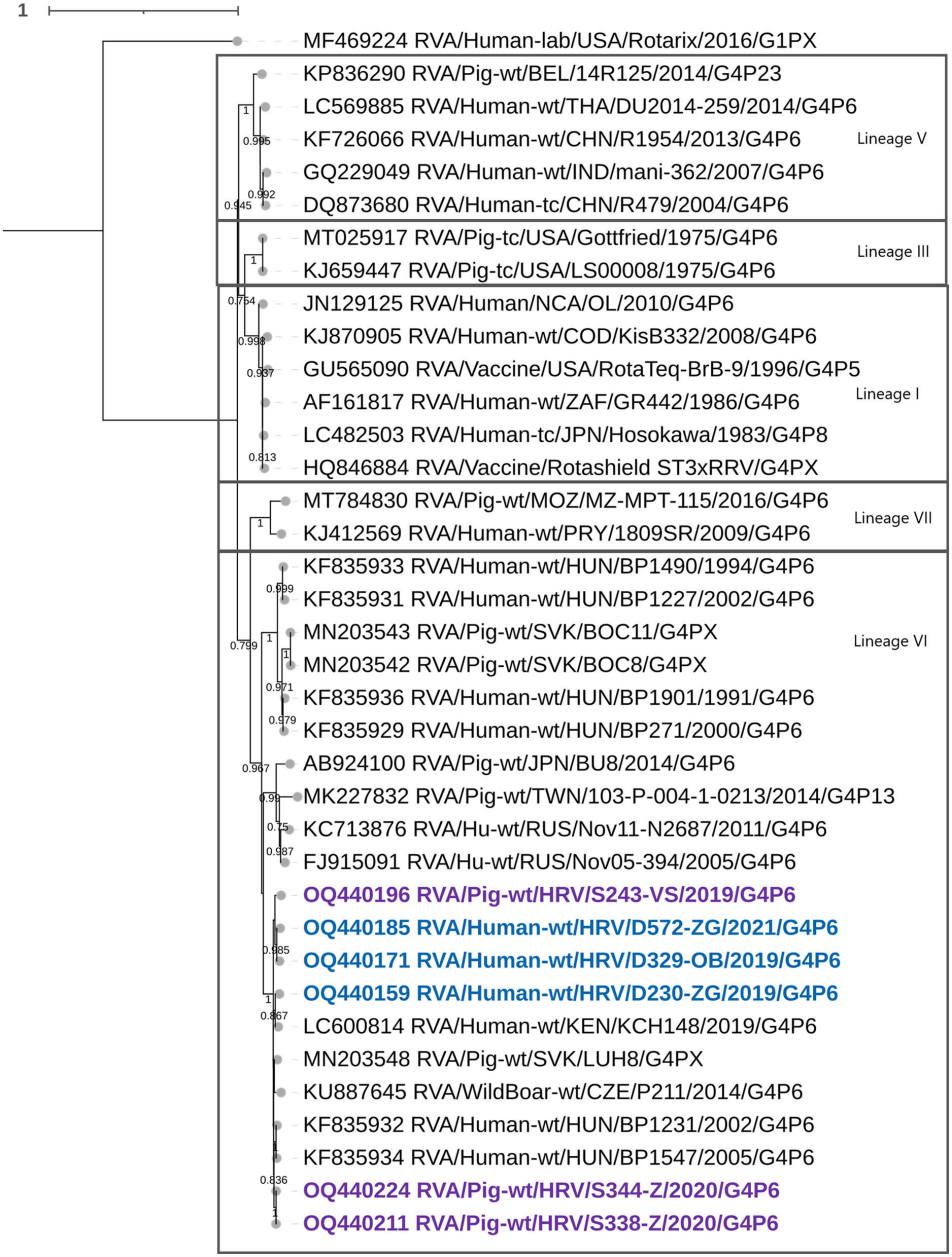
The phylogenetic tree of the full-length VP7 segment G4 genotype CDS sequences. The strains from the present study were bolded and marked in purple (for pig-derived strains) and blue (for human-derived strains). Accession numbers of all strains are included in the taxa labels. The tree was generated by the ML method and T92+G+I model in MEGA 11 software. The bootstrap analysis with 1,000 replicates was used to assess the branching support (showed values > 0.7). The scale bar represents the number of substitutions per site. Rotarix G1 strain as an outgroup.

Croatian G4 strains presented in this research belonged to lineage VI (Figure 1), which shared an nt percentage identity of approximately 83–86% with other G4 lineages. Intralineage nt percentage identity was larger than 86%. Our G4 strains (both porcine and human detected) formed a separate cluster within lineage VI with two porcine-derived strains from the Czech Republic and Slovakia and three G4 zoonotic strains detected in humans in Hungary and Kenya (Figure 1). Sequences in this cluster shared high nt similarities (94–100%), revealing that the three human-derived G4 Croatian strains have a porcine origin. Phylogenetic analysis for mixed genotypes that occurred in the VP7 segment, listed in Table 2, can be found in Supplementary Figure 1A. In addition, respective nt and aa % identities are located in Supplementary Table 3. The RDP4 recombination analysis detected no intragenotype or intergenotype recombination events in this gene segment.

#### 3.2.2. VP4

**Figure 2:**
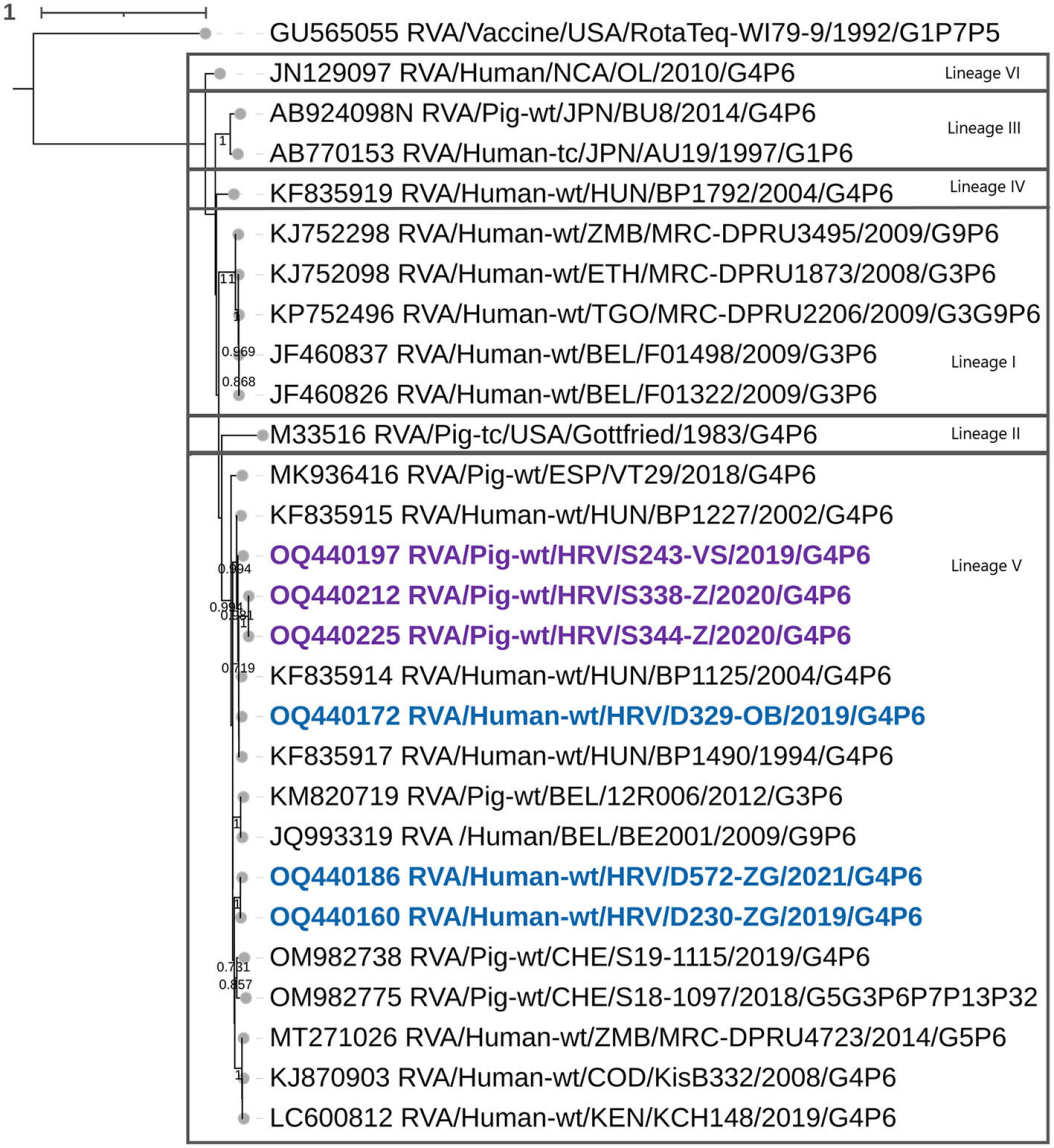
The phylogenetic tree of the full-length VP4 segment P[6] genotype CDS sequences. The strains from the present study were bolded and marked in purple (pig-derived strains) and in blue (human-derived strains). Accession numbers of all strains are included in the taxa labels. The tree was generated by the ML method and the HYK+G+I model in MEGA 11 software. The bootstrap analysis with 1,000 replicates was used to assess the branching support (showed values > 0.7). The scale bar represents the number of substitutions per site. RotaTeq P[5] strain stands as an outgroup.

Phylogenetic analysis of the VP4 segment grouped Croatian P[6] strains within the lineage V, among the zoonotic P[6] strains from Hungary, multiple zoonotic P[6] African strains, and P[6] strains detected in European pigs (Figure 2). Three Croatian pig RVA strains formed a separate clade, human-detected strains also, all of which shared the greatest nt similarity within their respective clades, while the D329 P[6] strain displayed porcine origin closest to Hungarian zoonotic P[6] (Figure 2). Genotype P[6] displayed intragenotype differences much like the G4; hence, nt percentage identities between different lineages ranged from 82 to 88%, and within the lineage V from 90 to 99.6%. Recombination analysis of the VP4 segment resulted in identifying the porcine S338 strain as an intragenotype P[6] recombinant of two human P6 strains of zoonotic porcine origin, from Hungary and Russia as major and minor parents (Table 3). The recombination event has been detected with six of seven RDP4 detection methods, therefore, strongly supported. Phylogenetic analysis for mixed genotypes that occurred in the VP4 segment, listed in Table 2, and can be found in Supplementary Figure 1B. In addition, respective nt and aa % identities are located in Supplementary Table 3.

**Table 3:** RVA intragenotype and intergenotype recombination data.

**Recombinant strain**	**S338 P6**	**D329 A1**	**D329 T7**	**S243 T7**	**D230 T1**
Recombination type	Intragenotype	Intragenotype	Intergenotype	Intergenotype	Intergenotype
Major parent	KF835917 RVA/Hu-wt/HUN/1490/1994/ G4P[6]	KF835940 RVA/Hu-wt/HUN/BP1231/2002/ G4P[6]A1	KF723308 RVA/Pig-wt/ITA/519RE/2010/ G5P23T7	D572 T7	OM982754 RVA/Pig-wt/CHE/S18-1463/2018/ G9P[32]T1
Minor parent	JX156399 RVA/Hu-wt/RUS/N2687/2011/ G4P[6]	ON992465 RVA/Hu-wt/CHN/JL18221043/ 2018/G9P[8]A1	D329 T1	S243 T1	D230 T7
Starting breakpoint^*^	1,858	885	246	1/838	310
Ending breakpoint^*^	2,156	971	575	174/943	692
No. of detection methods confirming recombination event	6/7	7/7	7/7	7/7	7/7

^*^Breakpoint confidence interval 99%.

#### 3.2.3. VP6

**Figure 3:**
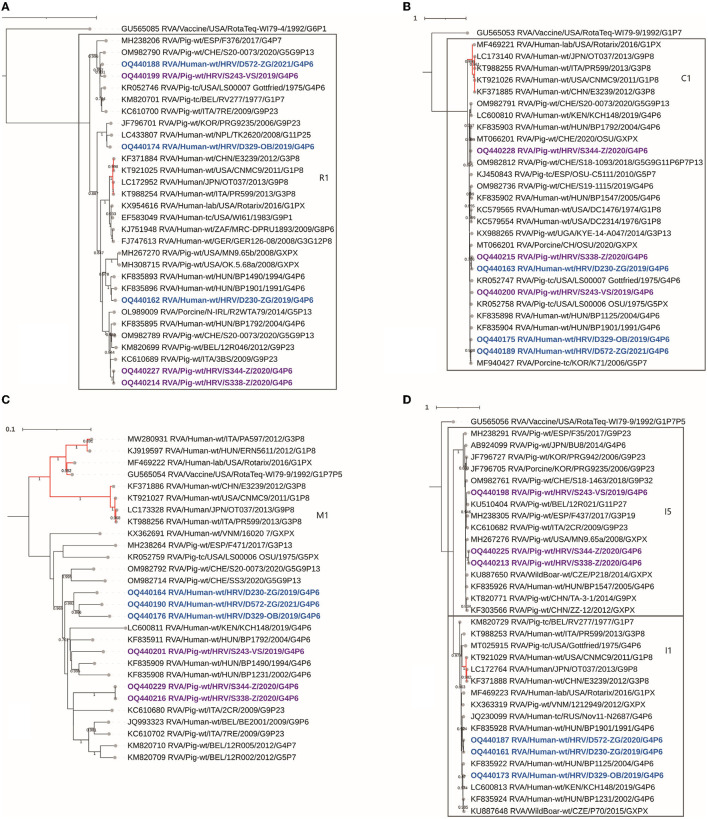
The phylogenetic tree of the full-length backbone viral proteins CDS sequences: VP1 **(A)**, VP2 **(B)**, VP3 **(C)**, and VP6 **(D)**. The strains from the present study were bolded and marked in purple (pig-derived strains) and in blue (human-derived strains). Accession numbers of all strains are included in the taxa labels. The branches colored in red are designated to typically human RVA strains to accentuate the separation between porcine and human-originated RVAs of the same genotype. The tree was generated in MEGA 11 software by the ML method, models TN93+G+I, and T92+G, respectively. The bootstrap analysis with 1,000 replicates was used to assess the branching support (showed values > 0.7). The scale bar represents the number of substitutions per site. RotaTeq genogroup 2 strains stand as an outgroup, besides VP3 **(C)**, where it shares the M1 genotype.

Two different genotypes were established in this segment, I5 for domestic pig-derived, and I1 for human-derived strains (Figure 3D). Intragenotype I5 nt similarity fell in the range of 92.8–99.8%. Among the typical porcine I5 genotype, two clusters of different sources of origin can be recognized, one that includes strain S243 mixed with other European strains (from Italy, Belgium, and Spain) and one of remote origin that includes strains S338 and S344 mixed with North American strain (Figure 3D). Genotype I1 human-derived strains presented as porcine-originated, since these strains branch together with Hungarian and Kenyan porcine-like strains previously reported as zoonotic (Papp et al., 2013a; Wandera et al., 2021) with nt similarity surpassing 95%. The RDP4 recombination analysis detected no intragenotype or intergenotype recombination events in this gene segment.

#### 3.2.4. VP1

Six Croatian G4P[6] strains were identified as genotype R1, although they proved to be a diverse group of sequences with nt similarity ranging from 86 to 100%. Human-derived strain D572 clustered with various European porcine R1 strains (94–96% nt identity) including an autochthonous S243 domestic pig-derived strain, and with the USA Gottfried, a representative strain for the porcine-originated Wa-like G4P[6] constellation (Figure 3A). Strain D572 R1 did not prove similar to any human-derived R1 available sequences (< 87%). This complete phylogenetic separation from human-derived R1 strains suggests a VP1 porcine/porcine-like human reassortment event. The second human-derived R1 strain, D230, clustered with Hungarian zoonotic porcine-like strains (Figure 3A). Interestingly, the human-detected D329 strain also displayed a porcine-like origin but was similar to porcine and porcine-like R1 strains from South Korea and Nepal (Figure 3A). The remaining two porcine-detected strains (S344, S348) were mixed with other European porcine strains in a different R1 lineage (Figure 3A). The RDP4 recombination analysis detected no intragenotype or intergenotype recombination events in this gene segment.

#### 3.2.5. VP2

All presented Croatian strains genotyped as C1 shared a porcine or porcine-like origin. A few typical human strains with Wa-like backbone constellation, including the Rotarix vaccine strain, were added to phylogenetic analysis for comparison purposes, and have formed a separate cluster, thus outlining the phylogenetic distance between human and porcine-originated genotype C1 (Figure 3B). Two porcine-derived Croatian strains, S338 and S344, sampled at the same time and on the same holding, separated into different clusters in this gene segment (Figure 3B). What probably influenced this separation is the insertion of S amino acid at position 41 in the amino acid sequence for strain S338. Moreover, additional insertions were observed in other Croatian C1 sequences, such as NNKN amino acids at positions 38–41 for strains D329 and S243, and KAS amino acids at positions 39–40 for strain D230. Listed insertions were sequenced with the high coverage for each nt position. The typical genotype C1 strains of human origin shared an insertion similar to the strains D329 and S243, differing in two amino acids (KNRN). In contrast, the insertion described for the D230 strain has not been, to the best of our knowledge, described yet. Regardless of the mentioned differences, nt similarity among porcine- and human-derived C1 strains ranged from 93 to 99%. The RDP4 recombination analysis detected no intragenotype or intergenotype recombination events in this gene segment.

#### 3.2.6. VP3

Croatian strains shared an M1 genotype consistent with porcine or porcine-like origin. Croatian human-originated M1 sequences clustered separately as shown in Figure 3C but phylogenetically connected to porcine RVA strains. Domestic pig-derived strains S344 and S338 clustered separately from strain S243, but all were phylogenetically related to porcine or human RVA strains of porcine origin. All six strains described in the present study displayed the highest nt identity with Hungarian zoonotic porcine-like strains, but interestingly, except for the porcine-derived S338 and S344 strains mutually, none of the strains shared more than 94.3% similarity with another autochthonous or database-accessed strain. The said divergence of herein included M1 genotype strains was also suggested by the phylogram branching pattern and branch lengths (Figure 3C). The RDP4 recombination analysis detected no intragenotype or intergenotype recombination events in this gene segment.

#### 3.2.7. NSP1

**Figure 4:**
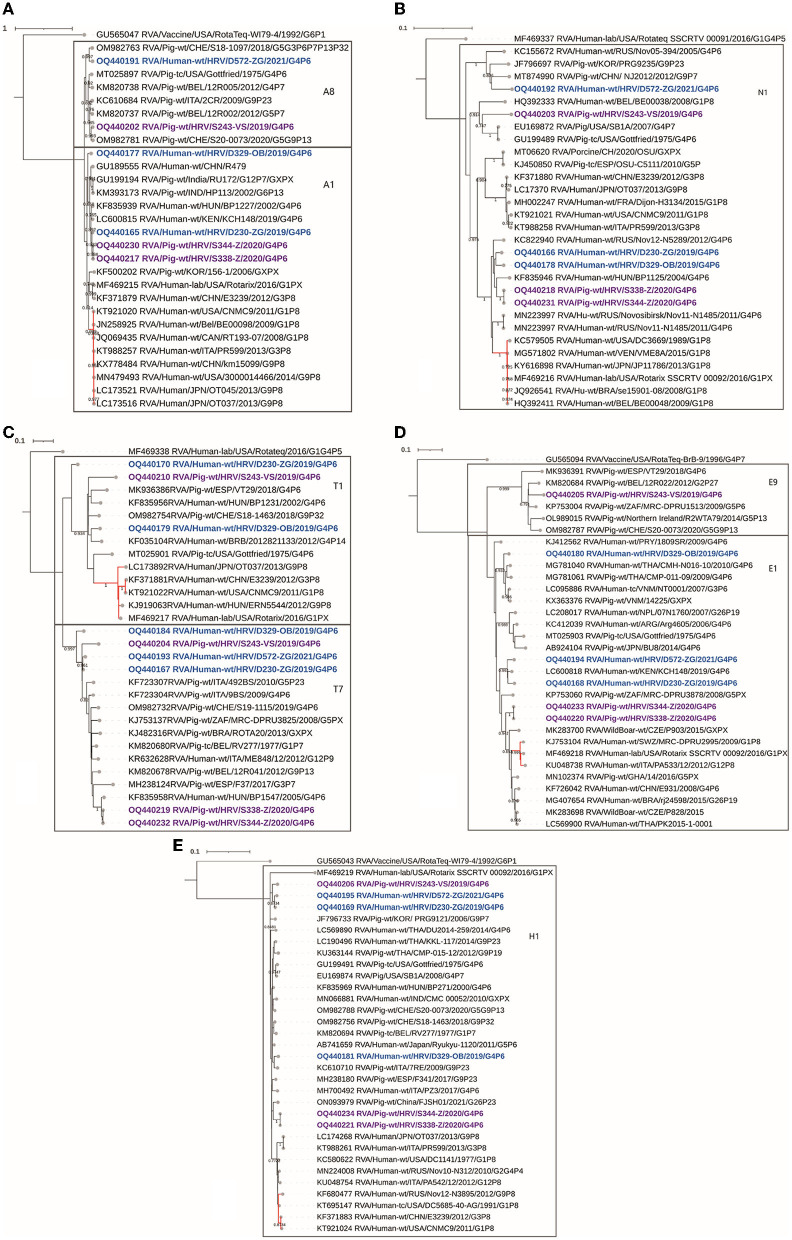
The phylogenetic tree of the full-length backbone non-structural proteins CDS sequences, NSP1 **(A)**, NSP2 **(B)**, NSP3 **(C)**, NSP4 **(D)**, and NSP5 **(E)**. The strains from the present study were bolded and marked in purple (for pig-derived strains) and blue (for human-derived strains). Accession numbers of all strains are included in the taxa labels. The branches colored in red are designated to typically human RVA strains to accentuate the separation between porcine and human-originated RVAs of the same genotype. The tree was generated in MEGA 11 software by the ML method, models T92+G+I **(A, C)** and T92+G **(B, D, E)**. The bootstrap analysis with 1,000 replicates was used to assess the branching support (showed values > 0.7). The scale bar represents the number of substitutions per site. RotaTeq genogroup 2 strains stand as an outgroup.

Within the NSP1 gene segment, the Croatian strains clustered into two genotypes, A1 and A8 (Figure 4A). A typical porcine A8 genotype was found in one porcine and one human-derived strain. The latter, the human-derived D572 A8 strain, was presented as a putative porcine/porcine-like human reassortment event, possessing a typical porcine genotype. Consequently, it separated phylogenetically with porcine strains, forming a clade with the porcine A8 strain detected in Switzerland (89.4% nt identity) supported by a high bootstrap value, while it barely reached the genotype cutoff value with other A8 strains. We accentuate the proximity of the Swiss porcine strain in this case since the reassortment suggests a putative evolutionary connection between D572 and porcine strains from Switzerland, which already occurred in the VP1 segment (Figure 3A). This finding marks a second reassortment event for the D572 strain, making it a porcine-human RVA reassortant in VP1 (Figure 3A) and NSP1 (Figure 4A). No available human-derived A8 sequences that would be similar to this strain were available in GenBank for comparison, pointing out a lack of known human-derived evolutionary relatives of the D572 A8 strain. In genotype A1, taxons branched in two separate directions, one of typical human-originated A1, and the other of porcine-like-originated A1. One human and two porcine Croatian sequences formed a clade in a porcine-like A1 cluster, while the human D329 strain branched individually within the same A1 cluster, with evident divergence, showing the highest nt identity of only 87.7% with a zoonotic porcine-like strain from Kenya (Figure 4A). The explanation for D329 A1 phylogenetic divergence was found in the NSP1 gene segment recombination analysis. It was identified as an intragenotype A1 recombinant between two human A1 strains, one of which was a zoonotic porcine-like origin strain from Hungary serving as a major parent, and in the role of the minor parent, there was a Chinese A1 strain of typical human origin (Table 3). This outcome is completely cohesive with the fact that in the D329 sample, along with the porcine-like G4P[6], G1P[8] genotype combination was also present, which is a typical human RVA genotype combination (Supplementary Figure 1). This recombination event was strongly supported by seven of seven RDP4 detection methods.

#### 3.2.8. NSP2

In the NSP2 phylogeny, Croatian strains were genotyped as N1 genotype, while clustering in three different branches (Figure 4B). One formed a cluster consisting of Croatian strains, two human and two porcine-derived, paired with a Hungarian zoonotic porcine-like strain. These four Croatian strains shared the highest percentage nt identity of approximately 97%, underlying an obvious connection between autochthonous porcine and porcine-like human strains. Furthermore, this cluster shared high identities at the nt level (>95%) with East Asian strains. Another strain, D572, also demonstrated phylogenetic proximity to far-eastern strains in a form of a clade with Chinese and South Korean pig strains (Figure 4B). The third putative source of origin was presented by two North American porcine N1 genotype strains, including Gottfried, in the same clade as the Croatian porcine S243 strain, as supported by pairwise nt identity comparison, and high bootstrap support (Figure 4B). The RDP4 recombination analysis detected no intragenotype or intergenotype recombination events in this gene segment.

#### 3.2.9. NSP3

The translation enhancer gene segment is presented in two genotypes, T7 and T1. In addition to VP7 and VP4 segments, mixed genotypes were also detected in NSP3 (Table 2, Figure 4C). All Croatian G4P[6] strains presented with typical porcine T7 genotype, grouping with other European and global porcine T7 strains (89–98%). Two human-derived strains (D230, D329) and one porcine strain (S243) were presented as a mix of T1/T7 genotypes. These strains shared the highest identity (92%) with other porcine and porcine-like human T1 strains (Figure 4C). In the T1 phylogram, the branches forming a clade with T1 RVA strains of human origin are highlighted in red to accentuate a divergence of porcine and porcine-like human strains detected in the present study. Moreover, the NSP3 segment headlined in recombination analysis, since every mixed genotype strain was also presented as an intergenotype T1–T7 recombinant (Table 3). This finding was also obvious in the phylogenetic tree as these strains occupied sequestered branches falling on the edges of their respective genotypes (Figure 4C). Human strain D329 T7 was profiled as a recombinant of the Italian porcine T7 strain (major parent) and D329 T1 (minor parent). Furthermore, human strain D230 T1 was detected to be a recombinant between a Swiss porcine T1 strain (major parent) and D230 T7 (minor parent). Finally, one more recombination event took place in the NSP3 segment, with a porcine strain S243 T7 as a recombinant, having S243 T1 as a minor, and another Croatian human porcine-like strain D572 T7 as a major parent (Table 3). Every listed recombination event was strongly supported because detection was achieved with six or seven RDP4 integrated detection methods.

#### 3.2.10. NSP4

Genome analysis of the RVA enterotoxin segment demonstrated two genotypes, E9 and E1. The porcine strain S243 presented with a typical porcine E9 genotype and is evidently related to a variety of European porcine strains (Figure 4D). The other five Croatian RVA strains described in the present study are positioned in the E1 genotype. Red-branching clade including the Rotarix vaccine strain shows the E1 genotype of human-origin RVAs (Figure 4D). Croatian E1 strains are positioned among porcine or porcine-like human strains in three different subclades, expressing high nt identity (92–100%) with autochthonous, European, African, Asian, and even Latin American strains, making it difficult to presume exact origin. The RDP4 recombination analysis detected no intragenotype or intergenotype recombination events in this gene segment.

#### 3.2.11. NSP5

All Croatian strains of human and porcine origin were genotyped as H1 genotypes showing over 97% nt identity among intragenotype strains (Figure 4E). Nevertheless, three clusters of Croatian H1 sequences can be determined phylogenetically. Two porcine sequences (S344, S388) branched with Chinese porcine H1 strain, one porcine (S243) and two human H1 porcine-like strains (D230, D572) sequestered in a separate clade, and finally, a human-derived porcine-like D329 strain branched with porcine H1 strain detected in Italy (Figure 4E). For origin reference, H1 strains of human RVA origin were marked in a red branching pattern and hence illustrated a separation from porcine-like H1 strains. The RDP4 recombination analysis detected no intragenotype or intergenotype recombination events in this gene segment.

## 4. Discussion

In the present study, we sequenced and analyzed the whole genomes of six Croatian RVA G4P[6] strains detected in children under 2 years of age with AGE symptoms and in weanling piglets with diarrhea, during a synchronized spatiotemporal 3-year study (2018–2021). The aim was to illustrate how genetically intertwined an unusual zoonotic G4P[6] RVA genotype can be in both populations concurrently, accentuating the influence that the animal rotaviruses have on the evolution and recurrence of heterotypic RVAs in humans. Expectedly, porcine RVA strains displayed to have a porcine genogroup 1 origin in all gene segments, with typical porcine genotypes such as I5, A8, T7, and E9 standing out. Three porcine-like human G4P[6] strains displayed a Wa-like genogroup 1 constellation, while phylogenetic analysis revealed that in every genomic segment, these strains were genetically closely related to porcine-like human RVAs or porcine-originated strains. Human RVA Wa-like genogroup constellation is known to share its origin with porcine RVA genogroup 1 strains (Matthijnssens et al., 2008b; Steyer et al., 2008; Martella et al., 2010; Papp et al., 2013b). Considering surface protein coding gene segments, the G4 genotype has also been proven to infect humans and pigs, predominantly as a part of the G4P[8] genotype combination in humans, and as a third most prevalent VP7 genotype in pigs (Doro et al., 2015). The same is accurate for P[6], which is also a major porcine genotype. Nevertheless, human porcine-like RVA P[6] strains have been identified in a very sporadic pattern in Europe, but recurrence was continuous (Bányai et al., 2004; Martella et al., 2006; Steyer et al., 2008; Papp et al., 2013a; Vrdoljak et al., 2019). In the present study, G4 genotype strains clustered within lineage VI as defined by Wandera et al. (2021). However, as we have already hypothesized, G4 lineage VI could actually be formed of three distinct lineages if the lineages I–V demarcation threshold was applied (Brnić et al., 2022), but the consensus threshold criterion for lineage definition is currently unknown. In actuality, all G4 lineage VI strains presented in that study branched into three groups, possibly marking different lineages (Brnić et al., 2022). Nevertheless, despite the linage notation, all human-derived G4 strains from the present study are of porcine origin (Figure 1). This could also be said for strains of the P[6] genotype which clustered within the lineage V (Figure 2). In our previous study on porcine RVAs in Croatia, two porcine P[6] strains clustered within lineage IV in addition to lineage V strains (Brnić et al., 2022). All these P[6] strains were closely evolutionary connected to neighboring Hungarian zoonotic P[6] strains, underlining the influence of regional geolocation on RVA strain diversity.

The timing of detection of human-derived G4[6] strains was uncommon as all three G4P[6] strains were detected in symptomatic children in the summer months, an RVA out-of-season period in Croatia. This comes in agreement with earlier reports that emphasize an increase in mixed and rare genotype rates in multiple European countries in out-of-season months (Hungerford et al., 2016). Similar findings were also reported in Southern Italy; a 6-month-old child infected with the zoonotic G4P[6] RVA strain paired with the Wa-like backbone constellation, was also hospitalized in August. The foreign origin of this strain was further hypothesized (Ianiro et al., 2019). Similar to neighboring Italy, Croatia is a Mediterranean country with an immense amount of tourism in July and August, thus, the import of an unusual zoonotic strain at that time could be hypothesized. However, based on the pairwise nt identities and phylogenetic relatedness of Croatian porcine and human-derived G4P[6] strains in the majority of gene segments, we believe that these cases are the result of independent events of indirect zoonotic interspecies transmission within Croatia. Moreover, the recombination analysis on multiple RVA segments provided additional evidence in favor of this conclusion. In all three human RVA cases, most probably an indirect RVA transmission occurred because of the very young age of infected children, where a direct piglet-child transmission is deemed highly unlikely. Environmental transmission might have played a role in the epidemiology of these infections. Since our human samples were collected during the summer months, the efficiency of RVA transmission might be reduced in higher temperature conditions (Kraay et al., 2018).

RVA mixed genotypes detected in Croatian porcine and porcine-like human G4P[6] strains propelled an incidence of reassortment events and intragenic homologous recombinations occurred in a few strains (Table 3). Due to the divergence of the D572 strain in VP1 and NSP1 segments from the rest of the human and porcine-like human strains, as well as clustering with exclusively porcine-derived strains in these segments, it most likely signifies the occurrence of reassortment between typical porcine and porcine-like human RVA strains (Figures 3A, 4A). No human-derived VP1 and NSP1 sequences that would be similar to the D572 strain were available in GenBank for comparison, pointing out a lack of known human-derived evolutionary relatives of D572 R1 and A8 strains, reaffirming D572 as a putative porcine/porcine-like human reassortant. It is accepted that heterologous RVAs of the porcine origin or porcine–human RVA reassortants had sporadically occurred and successfully spread among humans (Martella et al., 2010). Nevertheless, this kind of human-to-human transmission is generally short-lived since the heterologous RVA strains do not spread horizontally as efficiently among their non-specific hosts (Matthijnssens et al., 2006). Consequently, the significance of zoonotic transmission is potentially overlooked because clinically hospitalized symptomatic individuals are the focal point of RVA strain surveillance (Vilibic-Cavlek et al., 2021).

Moreover, two human porcine-like strains and one porcine strain have shown recombination events in at least one of the gene segments (VP4, NSP1 or NSP3). Interestingly, a G4P[6] RVA strain with a Wa-like constellation detected in the Dominican Republic was reported with the recombination events in the same genome segments as these three Croatian recombinants (Esona et al., 2017). Conversely to the comprehensive research of rotavirus A intragenic recombination prevalence, where recombination analysis of the NSP3 gene segment gave no results (Hoxie and Dennehy, 2020), herein we report T1–T7 intergenotype recombination among all three NSP3 recombinant strains, which also means that the NSP3 recombination was present in every strain presented with a T1/T7 mixed genotype. Findings like this further endorse the cognition that mixed genotypes predispose the evolution of novel RVA strains (Estes and Greenberg, 2013).

Finally, the VP2 nt sequence insertions in the 1–134 region are quite common (Matthijnssens et al., 2008a), and insertions detected in Croatian C1 sequences were found in the same region.

In conclusion, zoonotic interspecies transmission like these highlights the importance of continuous surveillance of animal RVAs and raises awareness on the role of animal RVAs in the evolution of strains affecting the human population. Such events of zoonotic transmission may have a short-term and long-term impact on the protectiveness of currently available vaccines. Thus, it is important to monitor the possible emergent liabilities which stem from the interconnection of human–animal RVAs. In that process, a One Health approach in RVA research brings an immense contribution.

## Data availability statement

The data presented in the study are deposited in the NCBI GenBank repository. Accession numbers are listed in Material and Methods and in the Supplementary Table 4.

## Ethics statement

The studies involving human participants were reviewed and approved by Board of Ethics of the University Hospital for Infectious Diseases Dr. Fran Mihaljević Zagreb; Board of Ethics of the Clinical Hospital Center Osijek; and Board of Ethics of the Institute for Public Health of Osijek-Baranja County. Written informed consent to participate in this study was provided by the participants' legal guardian/next of kin. The animal study was reviewed and approved by Board of Ethics of the Croatian Veterinary Institute.

## Author contributions

VKu and DB contributed to the conception and design of the study. SŠ, GT, VKo, and IRK provided human samples, assessed the Vesikari score, and collected the guardian's or next of kin's informed consent. TM and RK performed NGS runs. VKu and TK organized the database and conducted NGS data analysis. VKu wrote the first draft of the manuscript. RK, DB, AS, MP, and LJ wrote sections of the manuscript. DB was responsible for funding acquisition and project administration. All authors contributed to the manuscript revision, read, and approved the submitted version.

## Footnotes

^1^https://www.eurorotanet.com/project-information/documents-and-methods/ (accessed April 20, 2022).

^2^https://www.ncbi.nlm.nih.gov/genomes/VirusVariation/Database/nph-select.cgi?taxid=28875 (accessed January 9, 2023).

^3^https://blast.ncbi.nlm.nih.gov/Blast.cgi (accessed December 14, 2022).

^4^https://www.bv-brc.org/ (accessed December 20, 2022).

^5^https://rega.kuleuven.be/cev/viralmetagenomics/virus-classification/rcwg (accessed December 16, 2022).

^6^https://itol.embl.de/ (accessed February 24, 2023).

## Supplementary material

Supplementary Table 1 Reference-mapping data.

Supplementary Table 2 Nucleotide and amino acid percentage identity data.

Supplementary Table 3 Nucleotide and amino acid percentage identity data for VP7 and VP4 mixed genotypes.

Supplementary Table 4 GenBank accession numbers of deposited sequences.

Supplementary Figure 1 The phylogenetic tree of the detected mixed genotypes G1, G4, G5, G11 in the VP7 **(A)** and P[6], P[8], P[13] in the VP4 **(B)** gene segments. The strains from the present study were bolded and marked in purple (pig-derived strains) and in blue (human-derived strains). Accession numbers of all strains are included in the taxa labels. The tree was generated by the ML method, and T92+G+I model in MEGA 11 software. The bootstrap analysis with 1000 replicates was used to assess the branching support (showed values > 0.7). The scale bar represents the number of substitutions per site.
