# Supplementary figures and images for: Interspecies transmission of porcine-originated G4P[6] rotavirus A between pigs and humans: a synchronized spatiotemporal approach
Source: Front Microbiol. 2023 May 22;14:1194764. doi: 10.3389/fmicb.2023.1194764 (PMC10239803; doi:10.3389/fmicb.2023.1194764)

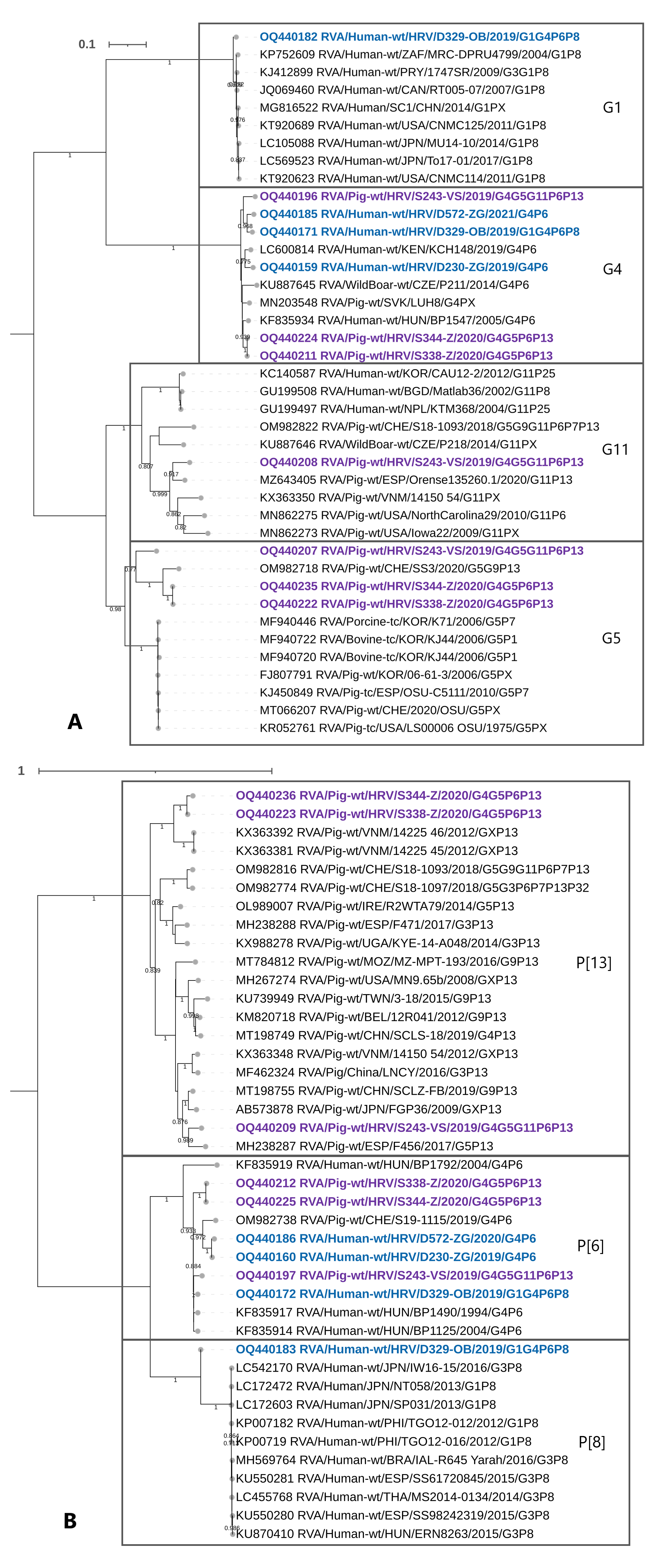

Supplement: Supplementary Figure 1 — The phylogenetic tree of the detected mixed genotypes G1, G4, G5, G11 in the VP7 (A) and P[6], P[8], P[13] in the VP4 (B) gene segments. The strains from the present study were bolded and marked in purple (pig-derived strains) and in blue (human-derived strains). Accession numbers of all strains are included in the taxa labels. The tree was generated by the ML method, and T92+G+I model in MEGA 11 software. The bootstrap analysis with 1000 replicates was used to assess the branching support (showed values > 0.7). The scale bar represents the number of substitutions per site. [file Image_1.TIFF]
